# Integrated Transcriptomic and Metabolomic Analyses Reveal Early Concentration-Dependent Responses of *Astragalus membranaceus* var. *mongholicus* Seedlings to Imidacloprid

**DOI:** 10.3390/genes17080891

**Published:** 2026-07-29

**Authors:** Dabao Yin, Xue Li, Li Zhou, Zhongchun Xiao

**Affiliations:** Qianxinan Collaborative Innovation Center for Characteristic Industrial Poverty Alleviation and Ecological Restoration, College of Biology and Chemistry, Minzu Normal University of Xingyi, Xingyi 562400, China; yindabao717@163.com (D.Y.); 13984663513@163.com (X.L.); zhouli@xynun.edu.cn (L.Z.)

**Keywords:** imidacloprid, *Astragalus membranaceus* var. *mongholicus*, metabolomics, transcriptomics

## Abstract

Background: Imidacloprid, a widely used neonicotinoid insecticide, is routinely applied to control pests in *Astragalus membranaceus* var. *mongholicus*, a crucial medicinal herb producing Astragali Radix. However, the early short-term transcriptional and metabolic responses of its seedlings under imidacloprid gradient stress remain poorly characterized. Methods: In this study, 80-day seedlings were subjected to three foliar spray treatments: blank control (CK), the recommended imidacloprid concentration (2000-fold dilution, 475 mg·L^−1^), and an excessively high concentration (500-fold dilution, 1900 mg·L^−1^). Leaf samples were harvested 24 h post-treatment for untargeted ultra-high-performance liquid chromatography-tandem mass spectrometry (UPLC–MS/MS) metabolomics (6 biological replicates) and RNA-seq transcriptome sequencing (3 biological replicates). Results: The low- and high-dose treatments induced 1076 and 860 differential metabolites and 6818 and 7283 differentially expressed genes, respectively. Flavonoids, saponins, terpenoids, amino acid metabolites, and energy-related pathways were prominently affected. KEGG enrichment indicated activation of flavone/flavonol biosynthesis, phenylpropanoid metabolism, amino acid metabolism, MAPK signaling, cutin/suberin/wax biosynthesis, and ABC transporter pathways, whereas high-dose exposure was associated with stronger changes in genes related to DNA replication and cell wall remodeling. Integrated network analysis highlighted *CHS*, *PAL*, *MYC2*, *KCS*, and *ABCG40* as candidate regulators linking stress signaling, secondary metabolism, and metabolite transport. Conclusions: Seedlings of *A. membranaceus* var. *mongholicus* exhibit dose-dependent acute responses to imidacloprid. Moderate pesticide exposure primarily activates defensive secondary metabolism, whereas excessive dosage triggers genome-wide transcriptional reprogramming. This work identifies key metabolic pathways and hub genes, offering candidate molecular markers for investigating pesticide stress adaptation in medicinal *Astragalus* and guiding standardized pesticide application in cultivation.

## 1. Introduction

*Astragalus membranaceus* var. *mongholicus*, a perennial leguminous herb, is one of the canonical botanical sources of Astragali Radix recorded in the Pharmacopoeia of the People’s Republic of China [1]. This species is naturally distributed across arid and semi-arid zones of northern China, including Inner Mongolia, Gansu and Shanxi Provinces [2,3]. Its dried root contains characteristic bioactive constituents, mainly astragalosides, flavonoids, and polysaccharides, which confer well-documented tonic, diuretic and tissue-repairing pharmacological activities [4,5]. Owing to overharvesting-induced shrinkage of wild populations, *A. membranaceus* var. *mongholicus* has been listed as a Class II national protected wild plant in China, and large-scale artificial cultivation has entirely replaced wild collection for industrial raw material supply [6]. In intensive artificial cultivation of *A. membranaceus* var. *mongholicus*, frequent infestations by piercing-sucking pests (aphids, thrips) significantly compromise root yield and the accumulation of bioactive constituents, so pest control measures become an indispensable part of field management [7,8].

Imidacloprid, a systemic neonicotinoid insecticide with high efficacy and low mammalian toxicity, has been widely applied to over 140 crop species worldwide for piercing-sucking pest management [9]. In commercial *Astragalus* plantations, foliar spraying of imidacloprid is the dominant strategy to suppress aphid and thrip infestation. Nevertheless, improper overdosage and repeated spraying impose abiotic chemical stress on non-target medicinal seedlings, disrupting plant photosynthesis, antioxidant homeostasis and root developmental programs [10,11]. Moreover, residual imidacloprid in harvested roots severely compromises medicinal material safety and restricts the market circulation of herbal products [12,13,14]. Previous physiological investigations on horticultural crops (pepper, pakchoi) have verified that high-concentration imidacloprid suppresses growth and disturbs core secondary metabolic pathways [15,16]. For *A. membranaceus* var. *mongholicus*, existing omics and physiological studies have characterized its adaptive responses to drought, salinity and fungal pathogen stresses [17,18,19], whereas the acute molecular and metabolic perturbations triggered by gradient imidacloprid exposure remain largely unelucidated [20].

Integrated transcriptomic and untargeted metabolomic profiling has become a robust paradigm to dissect plant abiotic stress regulatory networks, as it enables systematic linkage between transcriptional reprogramming and downstream metabolite accumulation [21,22,23]. Transcriptome sequencing identifies stress-responsive differentially expressed genes (DEGs) and signaling cascades, while metabolomics quantitatively captures dynamic shifts in primary and secondary metabolites that directly correspond to phenotypic stress symptoms. This multi-omics combinatorial strategy has been successfully deployed to decode pesticide-induced metabolic disorders in cereal and vegetable crops such as rice and chives, revealing coordinated alterations in carbohydrate, amino acid and lipid metabolism [24,25]. In medicinal plant research, however, no published study has combined transcriptomic and metabolomic approaches to characterize the 24 h acute leaf responses of *A. membranaceus* var. *mongholicus* under recommended vs. excessive imidacloprid concentrations, representing a critical knowledge gap in herbal pesticide stress biology.

To fill this research gap, the present study employed 80-day-old *A. membranaceus* var. *mongholicus* seedlings as experimental materials and established two imidacloprid treatments: the recommended field dilution (2000-fold, 475 mg·L^−1^, Low_RC) and an excessive overdosage (500-fold, 1900 mg·L^−1^, High_RC), with deionized water spraying as the blank control (CK). Leaf tissues were harvested at 24 h post-spray for RNA-seq transcriptome and UPLC–MS/MS untargeted metabolome detection. We systematically screened DEGs and differential metabolites and performed KEGG and GO enrichment, as well as multi-omics co-expression network analyses to identify core stress regulatory modules and hub genes. This work aims to (1) reveal the concentration-dependent early adaptive mechanisms of medicinal *Astragalus* seedlings under imidacloprid acute stress at the transcriptional and metabolic layers; (2) enrich the theoretical framework of abiotic pesticide stress tolerance in leguminous medicinal herbs; (3) provide molecular evidence to optimize standardized imidacloprid application protocols and guarantee the intrinsic quality and residue safety of cultivated Astragali Radix.

## 2. Materials and Methods

### 2.1. Plant Material, Cultivation and Pesticide Treatment

#### 2.1.1. Seedling Cultivation

Certified seeds of *A. membranaceus* var. *mongholicus* were utilized in this experiment. The authentic germplasm was collected from Hohhot, Inner Mongolia, China, and all seeds were healthy, pure, and pest-free, harvested in the autumn of the same year. Seed dormancy was disrupted mechanically via sandpaper abrasion, followed by a 1 h immersion in warm distilled water. Homogeneous plump seeds were sown in plastic pots filled with a standard nutrient substrate, with 12 seedlings planted per pot. All plants were cultivated in a programmable growth chamber under constant conditions: 25 ± 2 °C, 60–70% relative humidity, 14 h light/10 h dark photoperiod, and a photosynthetic photon flux density of 400 μmol·m^−2^·s^−1^. After 80 days of cultivation, seedlings with consistent height (20–25 cm) were retained, and uneven individuals were discarded. A total of 27 homogeneous pots were randomly assigned to three treatment groups via random number generation, with 9 pots per group.

#### 2.1.2. Imidacloprid Foliar Spraying

95% imidacloprid technical powder (CAS: 105827-78-9, purity ≥ 95.0%, Yuanye Bio, Shanghai, China) was diluted with ultrapure deionized water to establish three treatments: CK (Blank control): an equal volume of deionized water foliar spray; Low_RC (Recommended concentration): 2000-fold dilution (475 mg·L^−1^, standard field application dosage); High_RC (Excessive concentration): 500-fold dilution (1900 mg·L^−1^, 4× the recommended dosage to simulate pesticide overuse). A YATO YT-82005 handheld sprayer was used for uniform leaf coverage, with a fixed application volume of 2 mL per pot. All spraying operations were conducted at the same time point to eliminate circadian variation.

#### 2.1.3. Sample Harvest and Storage

At 24 h post-spray, the second fully expanded compound leaf of each seedling was collected to unify developmental stage. Leaves from multiple seedlings were pooled as one biological replicate: metabolomics: 6 biological replicates per group (*n* = 6), 6 mixed leaves per replicate; transcriptomics: 3 biological replicates per group (*n* = 3), mixed leaf tissue split equally for two omics tests. All leaf samples were immediately snap-frozen in liquid nitrogen, transported on dry ice to the laboratory, and long-term stored at −80 °C until extraction.

### 2.2. Untargeted UPLC–MS/MS Metabolomics Profiling

#### 2.2.1. Metabolite Extraction and Preprocessing

Frozen leaf samples were vacuum freeze-dried (Christ Alpha 1–4 LDplus, Martin Christ, Germany) and ground into fine powder with a pre-cooled mortar. Briefly, 50 mg of lyophilized powder of each replicate was extracted with 1 mL of extraction solvent (methanol: ultrapure water = 4:1, *v*/*v*) containing lidocaine (100 ppb, internal standard). The mixture was vortexed for 3 min, ultrasonicated at 4 °C for 30 min, then centrifuged at 12,000 rpm for 15 min at 4 °C. The supernatant was filtered through a 0.22 μm organic filter membrane, and 200 μL of filtrate was transferred to LC-MS vials for detection. QC quality control samples were prepared by mixing equal volumes of all 18 sample supernatants; one QC sample was injected every 10 runs to monitor instrument drift.

#### 2.2.2. Chromatography and Mass Spectrometry Parameters

Metabolite detection was performed by Luming Biotechnology (Shanghai, China) on a Waters ACQUITY UPLC I-Class system coupled with a Thermo Q Exactive Focus mass spectrometer (Thermo Fisher Scientific, Waltham, MA, USA). A BEH C18 column (2.1 mm × 100 mm, 1.7 μm, Waters, Milford, MA, USA) was adopted. Mobile phase A: 0.1% formic acid aqueous solution; Mobile phase B: 0.1% formic acid in acetonitrile. Gradient elution program: 0–1 min: 2% B; 1–9 min: linear gradient to 98% B; 9–12 min: hold 98% B; 12–13 min: linear gradient back to 2% B; 13–15 min: equilibration at 2% B. Flow rate = 0.3 mL·min^−1^, column temperature = 40 °C, injection volume = 2 μL.

MS acquisition mode: dual positive/negative ion mode. Spray voltage: 3.5 kV (ESI^+^), 3.2 kV (ESI^−^); capillary temperature = 320 °C; sheath gas flow = 40 arb. Full scan m/z range: 100–1000, resolution = 70,000. Secondary fragments were collected via data-dependent acquisition (DDA), with collision energy gradient set as 20/40/60 eV.

#### 2.2.3. Data Processing and Differential Metabolite Screening

Raw LC-MS data were preprocessed using Compound Discoverer 3.1 (Thermo Fisher, USA) for peak alignment, denoising, and preliminary annotation based on the in-house metabolite database of Luming Biotechnology. Processed data matrices were imported into R v4.2.2 for downstream analysis. PCA (Principal Component Analysis) was applied to evaluate intra-group repeatability and inter-group separation; OPLS-DA (Orthogonal Partial Least Squares Discriminant Analysis) combined with 7-fold cross-validation and 200 response permutation tests (RPT) was used to distinguish metabolic differences between groups. Models with R^2^Y > 0.9 and Q^2^ > 0.7 were regarded as valid to avoid overfitting. Differential metabolites (DMs) screening thresholds: VIP > 1.0 (OPLS-DA variable importance projection) + two-tailed Student’s *t*-test, adjusted *p* (FDR) < 0.05.

#### 2.2.4. Metabolite Annotation and KEGG Enrichment

Differential metabolites were annotated using the Kyoto Encyclopedia of Genes and Genomes (KEGG) database (Release 99.1), and enrichment analysis was performed using the clusterProfiler package in R. Emphasis was placed on metabolites associated with plant insect resistance, stress tolerance, and growth regulation (e.g., flavonoids, alkaloids, terpenoids, jasmonates, and amino acid derivatives). Key metabolites involved in the response of *A. membranaceus* var. *mongholicus* leaves to imidacloprid stress were preliminarily identified based on their expression trends and the significance of pathway enrichment.

### 2.3. RNA Isolation, Library Construction and RNA-Seq Transcriptome

#### 2.3.1. RNA Isolation and Quality Control

Total RNA was extracted from frozen leaf powder using the RNAprep Pure Plant Total RNA Kit (TIANGEN, Beijing, China; Cat. DP441) with on-column DNase digestion to eliminate genomic DNA contamination. RNA purity and concentration were measured on a NanoDrop 2000 spectrophotometer (Thermo Scientific, Waltham, MA, USA); RNA integrity number (RIN) was detected using an Agilent 2100 Bioanalyzer (Agilent Technologies, Santa Clara, CA, USA). Only samples with RIN ≥ 7.0, OD_260_/_280_ = 1.8–2.1, and OD_260_/_230_ > 1.8 were retained for library construction.

#### 2.3.2. cDNA Library Construction and Sequencing

RNA-seq libraries were constructed with the VAHTS Universal V6 RNA-seq Library Prep Kit (Vazyme, Nanjing, China). Qualified libraries were sequenced on the Illumina NovaSeq 6000 platform (Illumina, San Diego, CA, USA) to generate 150 bp paired-end reads. Sequencing and de novo assembly were completed by OE Biotech Co., Ltd. (Shanghai, China).

#### 2.3.3. Transcriptome Assembly and Gene Functional Annotation

Raw sequencing reads were trimmed with Trimmomatic v0.39 [26] to remove adapters, poly-N and low-quality reads, yielding clean reads. De novo transcript assembly was performed using Trinity v2.4 [27] with paired-end mode; the longest transcript of each gene cluster was defined as the unigene for subsequent quantification. Clean reads were mapped to unigenes via Bowtie2 v2.4.6 [28], and gene expression abundance was calculated as FPKM [29] values using eXpress v1.5.1 [30].

Unigene functional annotation was conducted via Blastx [31] (E-value ≤ 10^−5^) against seven databases: NR (2024), SwissProt (2024_03), KOG, KEGG Release 99.1 [32], Pfam v35, eggNOG, and GO. GO classification was mapped from SwissProt annotation information; KEGG pathways were assigned based on sequence homology.

#### 2.3.4. DEG Identification and Functional Enrichment

Differentially expressed genes (DEGs) were screened with DESeq v1.38 [33]. Screening criteria: adjusted *p*-value (q-value, BH FDR) < 0.05 and |log_2_(fold change)| > 1. Hierarchical clustering of DEGs was performed using Euclidean distance and complete linkage. GO and KEGG enrichment analyses were carried out based on the hypergeometric distribution, with BH correction to control the false discovery rate.

### 2.4. Integrated Transcriptome–Metabolome Multi-Omics Analysis

#### 2.4.1. Weighted Metabolite Co-Abundance Network (WMCNA)

All annotated target metabolites were subjected to weighted co-abundance network analysis with the R package WGCNA v1.71. Soft threshold power was automatically screened to satisfy the scale-free network topology. The Dynamic Tree Cut algorithm was used to divide metabolites into distinct co-expression modules. Module eigengene (ME) and gene significance (GS) values were calculated to quantify the correlation between each module and pesticide treatment phenotypes; core metabolites were defined as those with module membership kME > 0.9.

#### 2.4.2. WGCNA Gene Co-Expression & Module–Trait Correlation

Unigene expression matrices were imported into WGCNA v1.71 to construct gene co-expression modules. Spearman correlation coefficients were calculated between module eigengenes and three comparison phenotypes (Low_RC vs. CK, High_RC vs. CK, Low_RC vs. High_RC), with significance marked as * *p* < 0.05, ** *p* < 0.01, *** *p* < 0.001. Correlation heatmaps between gene modules and metabolite modules were visualized to reveal multi-layer regulatory relationships.

#### 2.4.3. Hub Gene Co-Expression Network Visualization

Five core regulatory genes (*CHS*, *PAL*, *MYC2*, *KCS*, *ABCG40*) were selected based on pathway enrichment results. Pairwise gene–gene and gene–metabolite Spearman correlations were calculated; pairs with |r| > 0.7 and *p* < 0.01 were retained for network construction. Networks were visualized using Cytoscape v3.9.1, with hub genes set as central nodes.

### 2.5. General Statistical Procedures

All statistical analyses were performed using R software (v4.2.2) and GraphPad Prism 9. For pairwise comparisons between two groups, two-tailed Student’s *t*-tests were applied. The Benjamini–Hochberg false discovery rate (FDR) method was used to correct *p*-values for multiple testing across all differential metabolite/gene screening and enrichment analyses. Pearson correlation was calculated for metabolite abundance profiles, while Spearman correlation was adopted to evaluate associations between gene/metabolite modules and phenotypic traits. Hierarchical clustering was conducted based on Euclidean distance with complete linkage agglomeration. Statistical significance was defined as: * *p* < 0.05, ** *p* < 0.01, *** *p* < 0.001.

## 3. Results

### 3.1. Untargeted Metabolomics Analysis

#### 3.1.1. Metabolite Profiling Analysis

**Figure 1:**
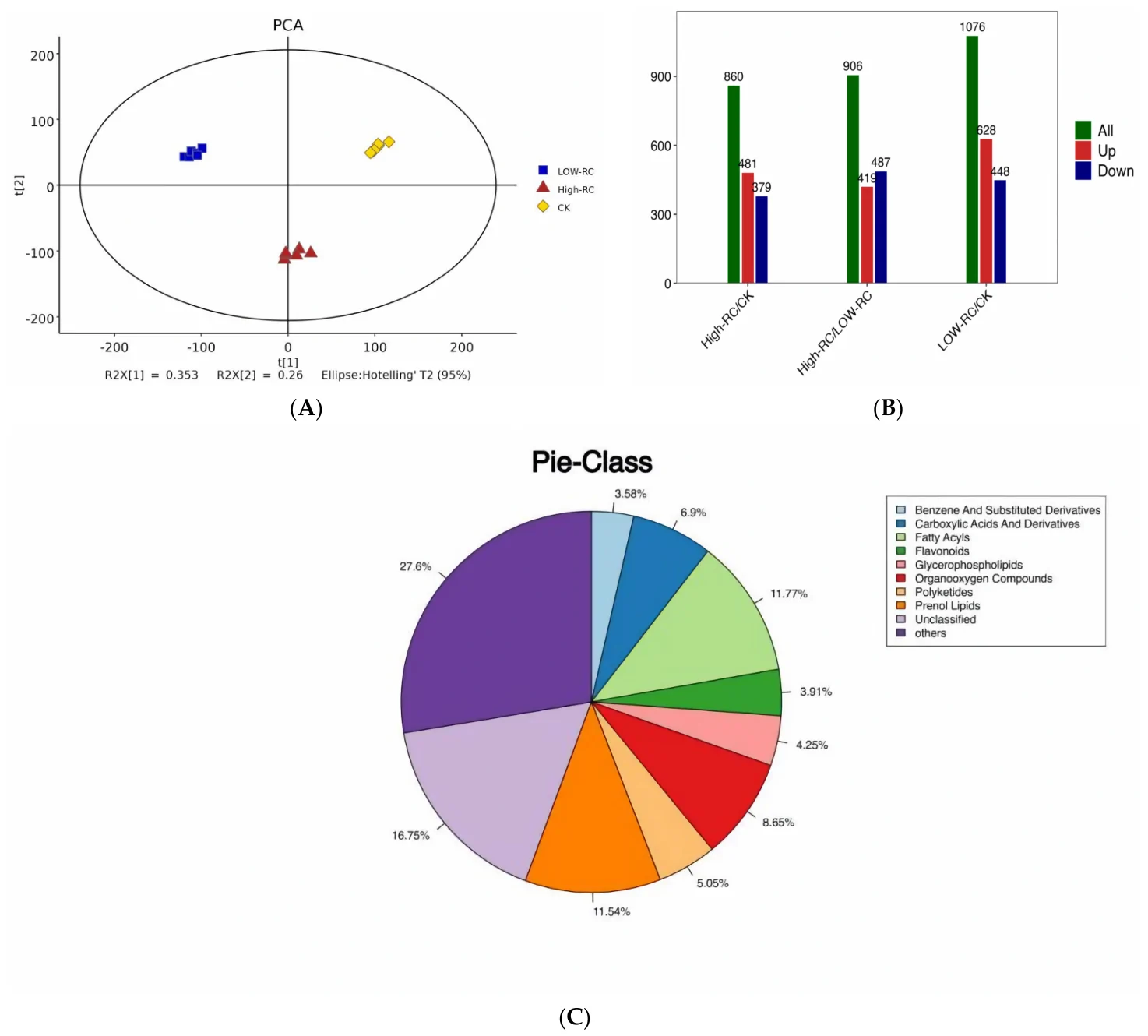
Overall analysis of metabolites: (**A**) metabolite main component analysis score chart; (**B**) statistics of the number of differential metabolites in each comparison group; (**C**) identify the category distribution of metabolites.

The results of principal component analysis (PCA) based on leaf metabolite data show that the first principal component (PC1) accounted for 35.3% of the total variance, while the second principal component (PC2) explained 26.0% of the variance, with a cumulative contribution rate of 61.3%. This indicated that these two principal components could effectively reflect the overall variation in the dataset. Samples from the same treatment group were closely clustered, demonstrating good intra-group repeatability. In contrast, distinct separations were observed among different treatment groups. Particularly along the PC1 axis, significant differences were detected between the control group (CK) and both the low-concentration imidacloprid group (Low_RC) and the high-concentration imidacloprid group (High_RC), suggesting that the treatment conditions exerted a significant impact on the metabolite composition. The 95% confidence ellipses indicated group separation (Figure 1A).

Statistical analysis of the number of differential metabolites (DMs) in each comparison group revealed the following: In the Low_RC vs. CK comparison, a total of 1076 DMs were identified, including 628 up-regulated and 448 down-regulated metabolites. In the High_RC vs. CK comparison, 860 DMs were detected, with 481 up-regulated and 379 down-regulated metabolites. Additionally, in the High_RC vs. Low_RC comparison, 906 DMs were identified, comprising 419 up-regulated and 487 down-regulated metabolites (Figure 1B). These results indicate that the low-concentration treatment induced the largest number of metabolite changes compared with the control group.

After metabolite annotation and identification, a total of 8185 metabolites were obtained and classified into different categories, namely: benzenoids and their derivatives (303), carboxylic acids and derivatives (583), acyl compounds (995), flavonoids (331), glycerophospholipids (359), organic oxides (731), polyketides (427), propenol lipids (976), other metabolites (2334), and unclassified metabolites (1416). The proportions of these categories in the total number of metabolites were 3.58%, 6.90%, 11.77%, 3.91%, 4.25%, 8.65%, 5.05%, 11.54%, 27.60%, and 16.75%, respectively (Figure 1C). These findings demonstrated that the leaf metabolites of *A. membranaceus* var. *mongholicus* were highly diverse. Among these, a considerable number of differential metabolites lacked definitive structural annotation and were categorized as unclassified metabolites. Owing to insufficient database matching information, biological interpretations were not conducted for this group of compounds.

#### 3.1.2. Hierarchical Clustering Analysis of Differential Metabolites

**Figure 2:**
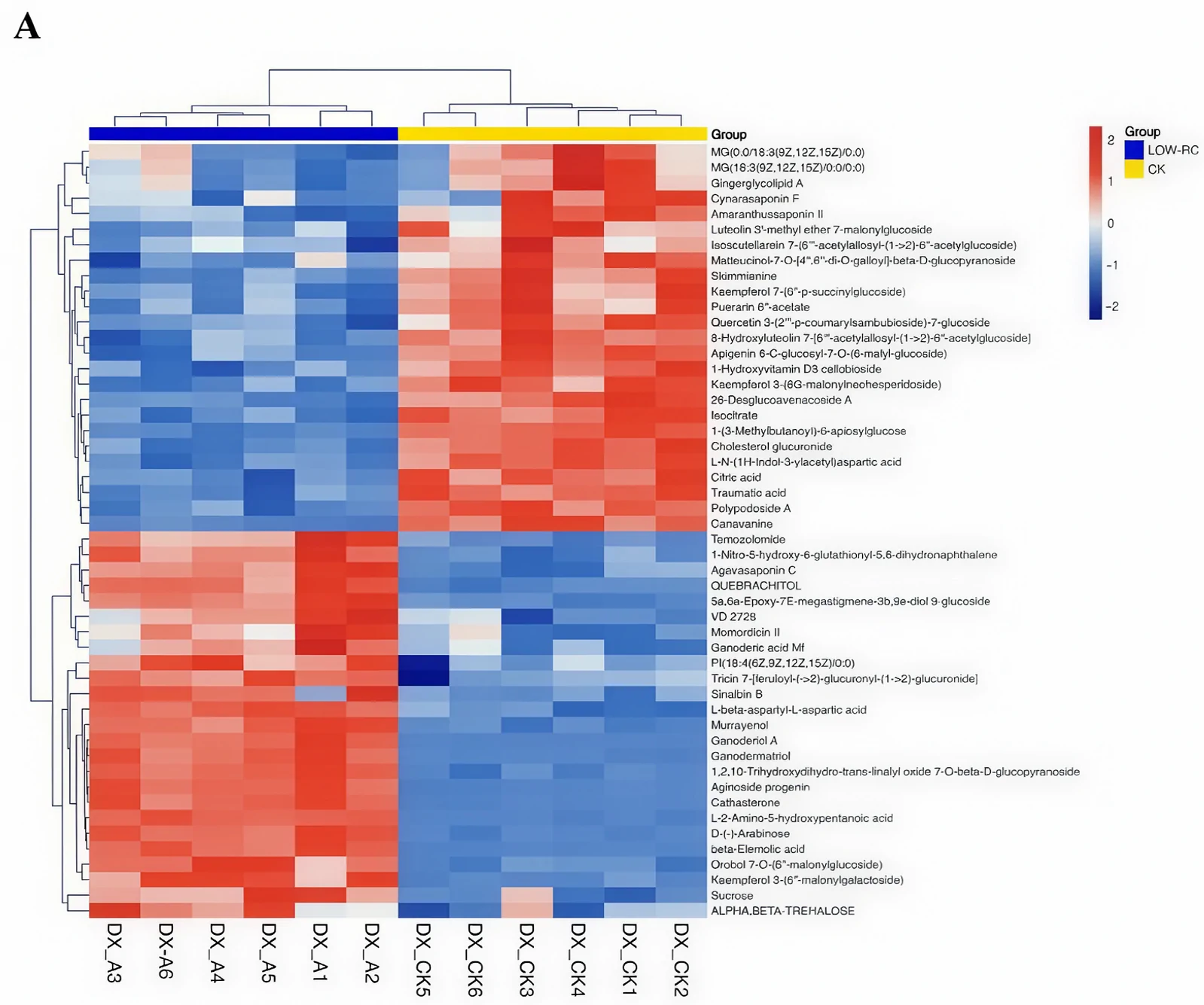

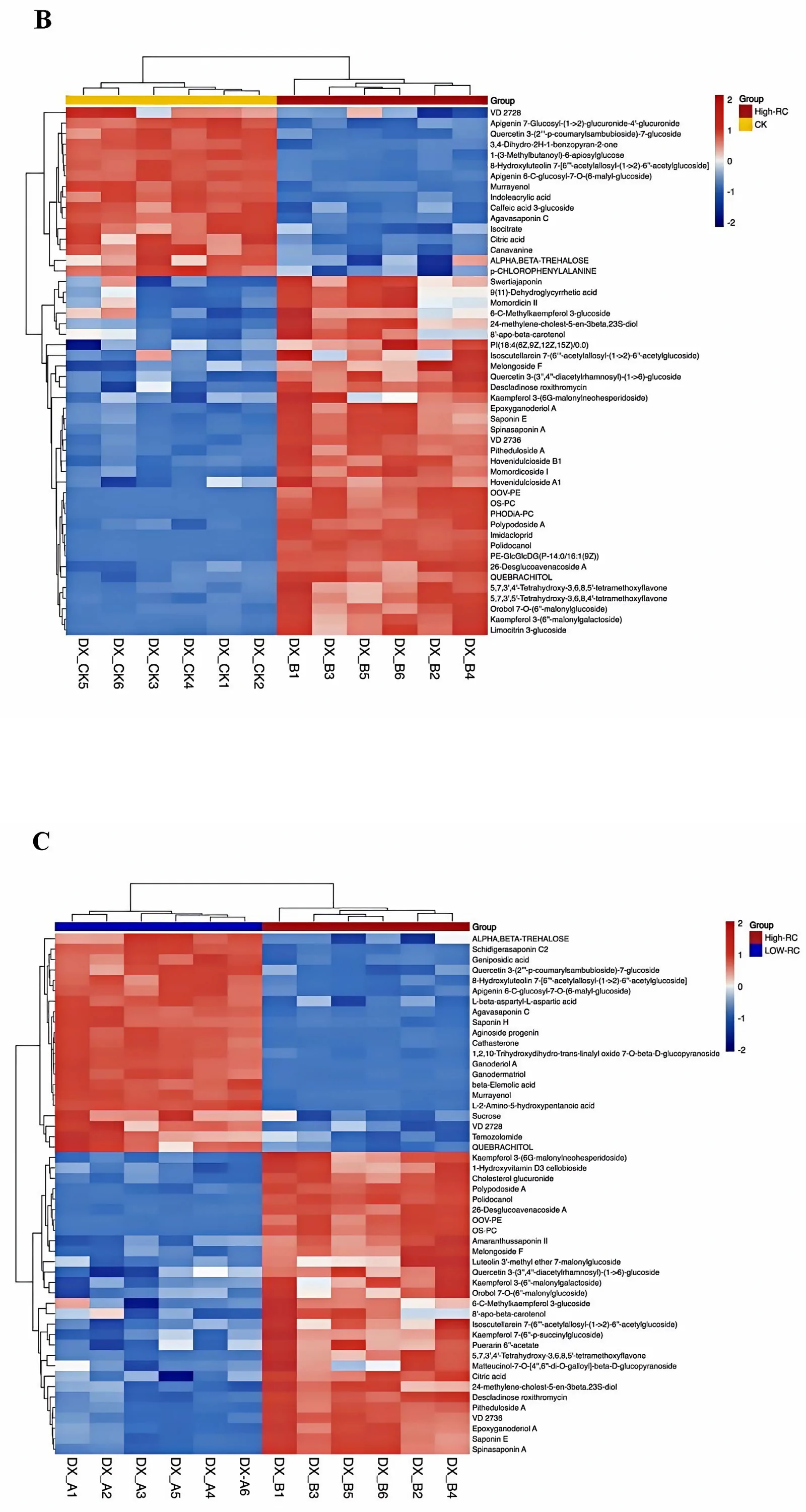
Cluster analysis of differential metabolites: (**A**) Low_RC vs. CK; (**B**) High_RC vs. CK; (**C**) High_RC vs. Low_RC (each subgraph shows the clustering heatmap of the differential metabolites in the corresponding comparison).

For the three comparison groups (Low_RC vs. CK, High_RC vs. CK, and High_RC vs. Low_RC), the top 50 most significantly altered differential metabolites (DMs) were extracted from each group for hierarchical clustering analysis. In total, 121 DMs with distinct variation patterns were identified (Figure 2A–C).

In the Low_RC vs. CK group, 41 DMs were identified (Figure 2A), which mainly included 11 flavonoids (e.g., luteolin 2-methyl ether-7-glucoside, kaempferol 7-acetylglucoside, etc.), 9 saponins (e.g., Cynatrasponin F, Amanifinisasponin H, Gnyanytychytol A, etc.), 6 glycolipids (e.g., monoglycerides and their derivatives, 1-(3-methylbutyryl)-6-O-palmitoylglucose, etc.), 5 amino acid derivatives (e.g., L-β-aspartyl-L-aspartic acid, L-2-amino-5-hydroxypentanoic acid, tetronic acid, etc.), and 7 terpenoids and sterols (e.g., cholesterol glucuronide, β-elemene acid, Polypodoxide A, etc.).

In the High_RC vs. CK group, 38 DMs were identified (Figure 2B), primarily consisting of 12 flavonoid derivatives (e.g., kaempferol 3-glucoside, astragalin diglucoside, etc.), 7 saponins (e.g., Agarasaponin C, saponin E, Monorotion II, etc.), 5 organic acids (e.g., citric acid, caffeic acid-3-glucoside, Inolateoxylic acid, etc.), and 9 terpenoids and lipids (e.g., 24-methylene-cholestene derivatives, 8′-apo-β-carotenol, Polypodostide A, etc.). The remaining metabolites were classified into other categories.

In the High_RC vs. Low_RC group, 42 DMs were identified (Figure 2C), mainly including 13 flavonoid glycosides (e.g., quercetin derivatives, kaempferol derivatives, luteolin methyl ether derivatives, etc.), 8 saponins (e.g., Agarasaponin C, saponin H, Schidgenraspornin C2, etc.), 4 carbohydrate derivatives (e.g., trehalose, stachyose, etc.), 7 terpenoids (e.g., β-elemene acid, Garacerene A, Carlasterone, etc.), and 5 phospholipids (e.g., COV-PC, OS-PC, PHODA-PC, etc.).

Combining the results of the above three comparison groups, a total of 121 DMs were identified, which were mainly categorized as follows: 36 flavonoids, 24 saponins, 23 terpenoids, 14 lipids and phospholipids, 10 organic acids and amino acid derivatives, and 10 carbohydrates/glycolipids. These results indicate that flavonoids, saponins, and terpenoids exhibited the most significant alterations in the leaves of *A. membranaceus* var. *mongholicus* in response to different imidacloprid treatments, highlighting the core roles of these secondary metabolites in plant stress responses.

#### 3.1.3. Pathway Enrichment Analysis of Differential Metabolites

**Figure 3:**
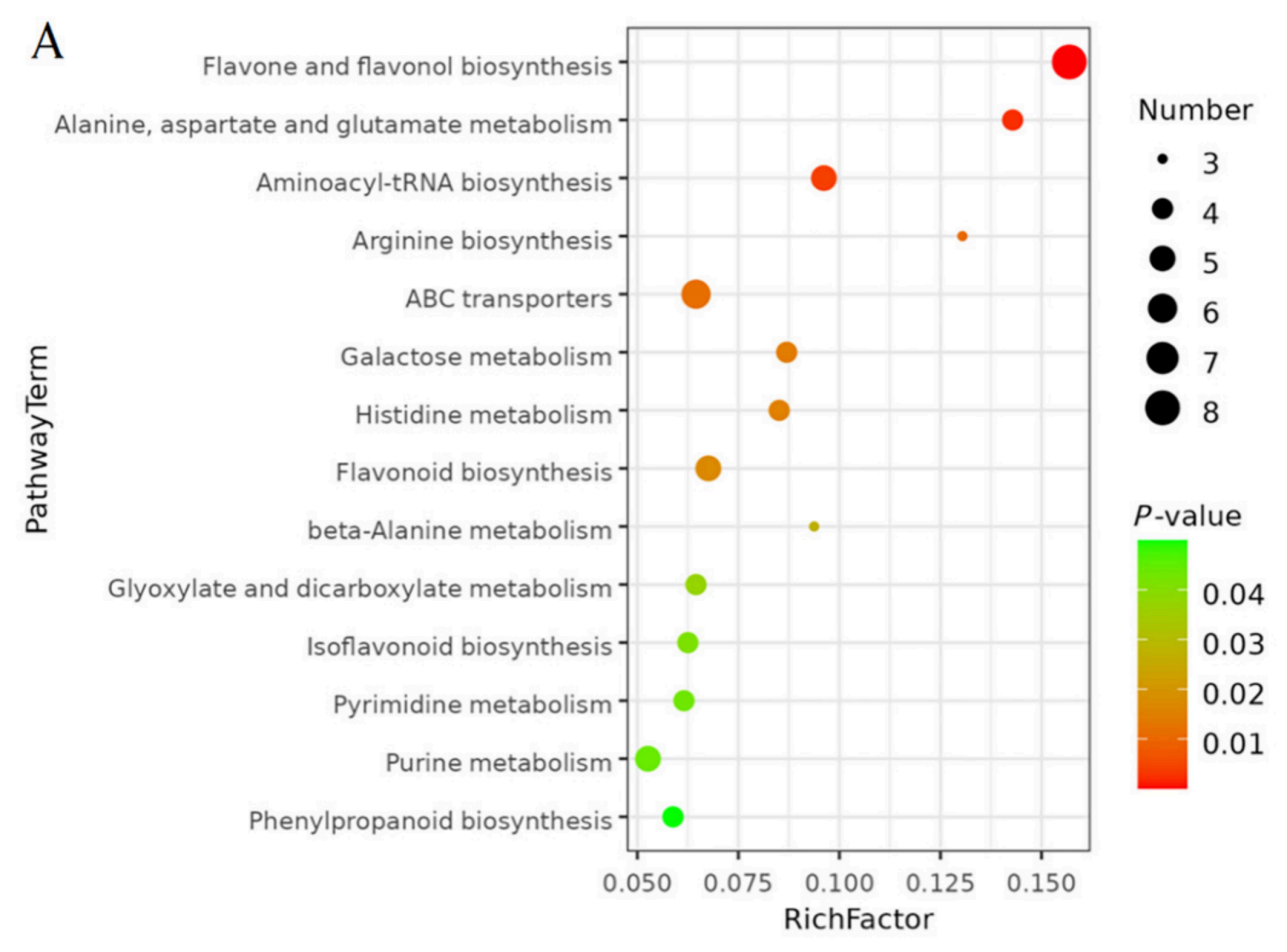

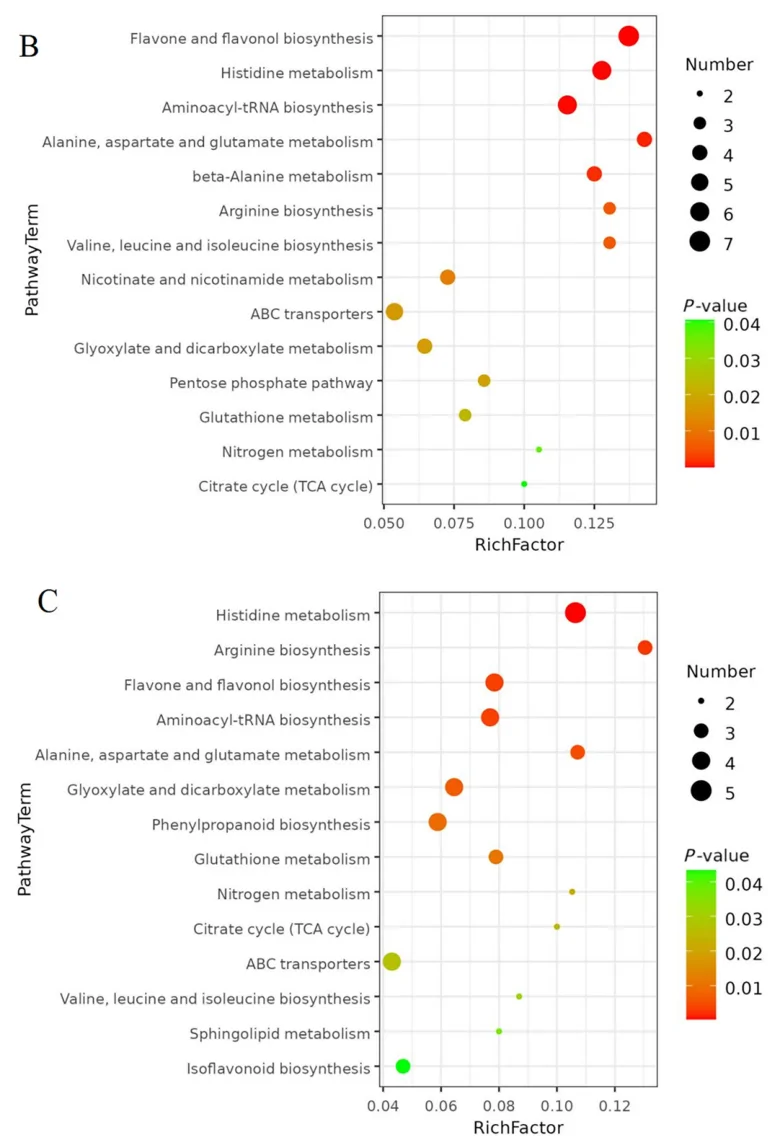
Pathway enrichment analysis of differential metabolites: (**A**) Low_RC-vs.-CK; (**B**) High_RC-vs.-CK; (**C**) High_RC-vs.-Low_RC (the bubble chart shows the significantly enriched KEGG pathways in each comparison group).

In the Low_RC vs. CK comparison group, differential metabolites (DMs) were significantly enriched in multiple metabolic pathways. These included common stress-responsive pathways such as flavone and flavonol biosynthesis, various amino acid metabolic pathways, and aminoacyl-tRNA biosynthesis, as well as a broader range of metabolic categories (Figure 3A). For instance, significant enrichment was observed in galactose metabolism, purine metabolism and pyrimidine metabolism, which implies that carbohydrate and nucleic acid metabolism levels may be up-regulated in seedlings, potentially providing substrates for cell proliferation and energy supply. Enrichment of the phenylpropanoid and isoflavonoid biosynthesis pathways suggests that secondary metabolism may participate in the plant response to imidacloprid stress. Moreover, enrichment was also detected in ABC transporters and β-alanine metabolism pathways, indicating that metabolic regulation may mediate stress responses via multiple functional layers, including carbon utilization, nucleotide synthesis and secondary metabolite accumulation.

In the High_RC vs. CK comparison group, DMs were significantly enriched in 14 KEGG metabolic pathways. These mainly included flavone and flavonol biosynthesis, histidine metabolism, aminoacyl-tRNA biosynthesis, and various amino acid metabolic pathways (e.g., alanine, aspartate and glutamate metabolism; arginine biosynthesis; and branched-chain amino acid biosynthesis) (Figure 3B). Furthermore, multiple energy metabolism pathways were also markedly enriched, including the tricarboxylic acid (TCA) cycle, pentose phosphate pathway, and glyoxylate and dicarboxylate metabolism. Enrichment of ABC transporters, glutathione metabolism and nitrogen metabolism pathways suggests that transmembrane transport, antioxidant reactions and nitrogen utilization may undergo prominent adjustments under high-dose pesticide stress. Collectively, these results imply that high-concentration imidacloprid exposure may trigger extensive metabolic reprogramming, which potentially affects core physiological processes ranging from secondary metabolism and biosynthesis to energy supply and stress defense.

In the High_RC vs. Low_RC comparison group, DMs were significantly enriched in 14 pathways. In addition to the repeatedly enriched pathways (e.g., histidine metabolism, arginine biosynthesis, flavonoid biosynthesis, and aminoacyl-tRNA biosynthesis), the enrichment of phenylpropanoid biosynthesis and isoflavonoid biosynthesis was particularly prominent. This indicated that high-concentration treatment activated the phenylpropanoid secondary metabolic pathways more strongly than low-concentration treatment (Figure 3C). Meanwhile, enrichment of sphingolipid metabolism implies possible modifications to membrane lipid composition and corresponding signal transduction. Repeated enrichment of glutathione metabolism, nitrogen metabolism and ABC transporter pathways suggests that plants may develop adaptive responses to varying imidacloprid concentrations by modulating antioxidant defense, nitrogen homeostasis and transmembrane substance transport.

### 3.2. Transcriptome Analysis

#### 3.2.1. Quality Assessment of Transcriptome Sequencing Data

The number of clean reads per sample ranged from 45.94 million (M) to 50.44 M, with the corresponding clean bases ranging from 6.71 gigabases (Gb) to 7.42 Gb. These results indicate that the sequencing depth was sufficient to support reliable transcriptome assembly and gene expression analysis. In terms of sequence quality, the Q30 percentage of all samples exceeded 90%. Among these, sample B3 exhibited the highest Q30 value (93.68%), while the remaining samples showed Q30 values ranging from approximately 92.8% to 93.5%, demonstrating high accuracy and good stability of the sequencing data. The GC content of each sample varied from 42.93% to 43.62%, which was consistent with that of typical plant transcriptomes and showed no significant bias (Table 1). A total of 54,748 Unigene sequences were obtained from transcriptome assembly, among which 19,067 sequences were functionally annotated, accounting for 34.83% of the total Unigenes (Table 2).

#### 3.2.2. Differentially Expressed Gene (DEGs) Analysis

**Figure 4:**
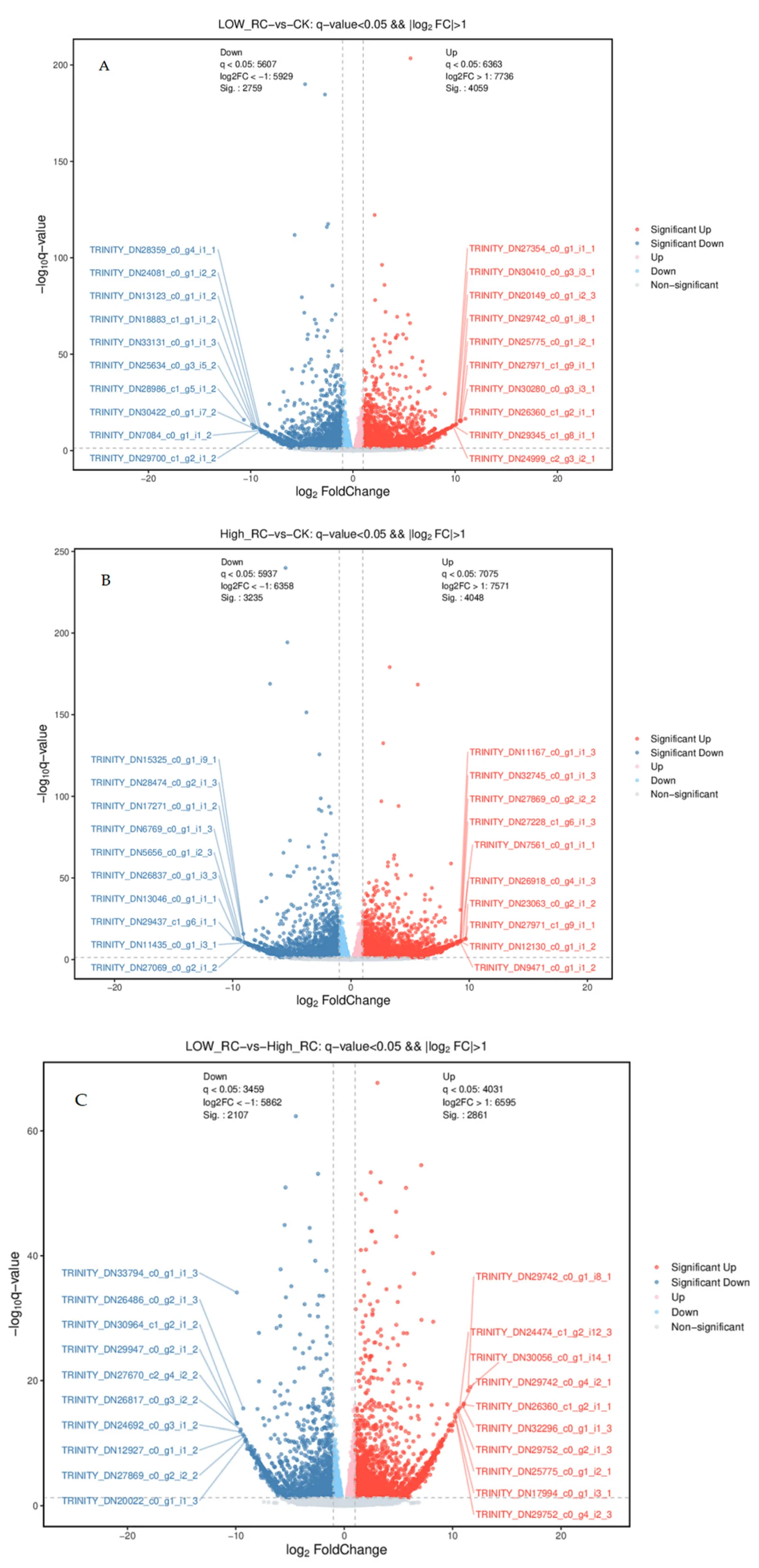
Statistics of differentially expressed genes in different comparison groups: (**A**) Low_RC-vs.-CK; (**B**) High_RC-vs.-CK; (**C**) Low_RC-vs.-High_RC (the numbers of up-regulated and down-regulated genes in each comparison group are in parentheses).

To investigate the molecular response mechanisms of *A. membranaceus* var. *mongholicus* under different concentrations of imidacloprid stress, pairwise comparisons were performed among the three treatment groups to screen for differentially expressed genes (DEGs). The results are presented in Figure 4A–C: In the Low_RC vs. CK comparison, a total of 6818 DEGs were identified with the screening criteria of q-value < 0.05 and |log_2_FC| > 1, including 4059 up-regulated genes and 2759 down-regulated genes (Figure 4A). In the High_RC vs. CK comparison, 7283 DEGs were detected, with 4048 up-regulated and 3235 down-regulated genes (Figure 4B). In the Low_RC vs. High_RC comparison, 4968 DEGs were identified, comprising 2861 up-regulated and 2107 down-regulated genes (Figure 4C). Overall, the high-concentration treatment group (High_RC vs. CK) exhibited the largest number of DEGs, indicating that *A. membranaceus* var. *mongholicus* triggered a more robust transcriptional response under high-dose imidacloprid stress.

Notably, the number of up-regulated DEGs was higher than that of down-regulated DEGs in all comparison groups, especially in the Low_RC vs. CK group. These transcriptional profiling changes indicated that imidacloprid stress substantially altered the gene expression patterns of *A. membranaceus* var. *mongholicus*, presenting a general upregulation tendency of numerous genes that may participate in the molecular response to pesticide stress. The detailed regulatory functions of these up-regulated genes still require further verification. The identified DEGs provide a fundamental dataset for subsequent functional annotation and pathway enrichment analysis, facilitating further exploration of the core transcriptional characteristics of *A. membranaceus* var. *mongholicus* under imidacloprid stress.

#### 3.2.3. GO Functional Annotation of Differentially Expressed Genes

**Figure 5:**
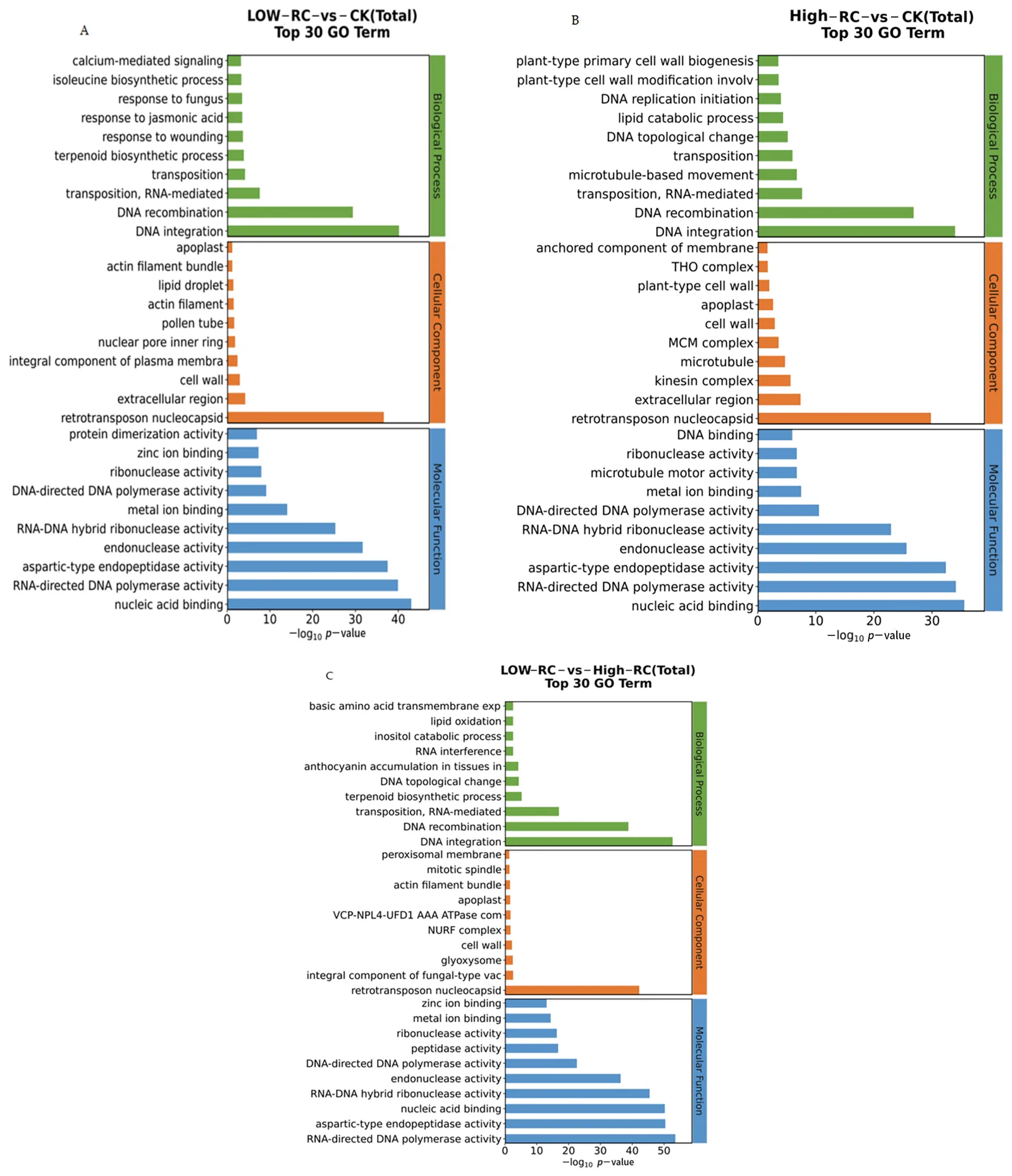
GO classification enrichment results of differentially expressed genes under different treatments: (**A**) Low_RC-vs.-CK; (**B**) High_RC-vs.-CK; (**C**) Low_RC-vs.-High_RC (the bar chart shows the enriched and significant GO entries, sorted by significance).

To elucidate the functional characteristics of differentially expressed genes (DEGs) in *A. membranaceus* var. *mongholicus* under different imidacloprid concentrations. Gene Ontology (GO) functional enrichment analysis was performed on the DEGs of each comparison group. The results of GO classification were presented in three major categories: biological process (BP), cellular component (CC), and molecular function (MF). Figure 5A–C show the top 30 significantly enriched GO terms in the three comparison groups, respectively.

In the Low_RC vs. CK comparison (Figure 5A), DEGs were significantly enriched in multiple GO terms related to stress response and primary metabolism. For example, the BP category included calcium-mediated signaling, isoleucine biosynthetic process, and response to jasmonic acid; the CC category was enriched in cell wall, integral component of plasma membrane, and extracellular region; the MF category comprised zinc ion binding, metal ion binding, and various nuclease activities such as RNA-directed DNA polymerase activity. These results indicate that low-dose imidacloprid mainly affected stress signal perception, basic metabolism, and cell wall structural composition in *A. membranaceus* var. *mongholicus*.

In the High_RC vs. CK comparison (Figure 5B), the enriched GO functional terms of DEGs were distinct. The BP category prominently featured plant-type primary cell wall biogenesis, DNA replication initiation, and transposition; the CC category was enriched in MCM complex, microtubule motor complex, and retrotransposon nucleocapsid; the significant terms in the MF category included DNA binding, microtubule motor activity, and various enzyme activities related to nucleic acid metabolism. Particularly noteworthy was the significant enrichment of both DNA-directed DNA polymerase activity and the aforementioned RNA-directed DNA polymerase activity. Since this study only relies on transcriptome enrichment trends without experimental verification of transposon activity and genomic stability, we refrain from further speculating on their biological functions. The relevant transcriptional differences can only be regarded as important characteristics of transcriptional perturbation in plants under high-concentration pesticide stress.

In the Low_RC vs. High_RC comparison (Figure 5C), the enriched GO terms of DEGs were relatively fewer and more scattered. The prominent terms in the BP category were basic amino acid transmembrane transport, lipid oxidation, and RNA interference; the CC category was enriched in peroxisomal membrane, mitotic spindle, and glyoxysome; the MF category included zinc ion binding, metal ion binding, and aspartic-type endopeptidase activity. Overall, the transcriptional differences between the high- and low-concentration treatments were mainly concentrated in certain specific metabolic regulations and organelle functions, such as lipid metabolism, antioxidant defense, and post-transcriptional gene silencing mechanisms, with a lower enrichment level compared with the other two groups.

In summary, different concentrations of imidacloprid significantly altered the transcriptomic functional characteristics of *A. membranaceus* var. *mongholicus*. DEGs in all three comparison groups were extensively enriched in functional categories related to catalytic activity, ion binding, nucleic acid metabolism, and cellular structure. High-concentration imidacloprid triggered more drastic functional reprogramming, involving typical stress response mechanisms such as cell wall reconstruction, DNA replication, and transposition activation. This indicated that high-dose pesticides exerted a deeper-level impact on plant cells.

#### 3.2.4. KEGG Pathway Enrichment Analysis of Differentially Expressed Genes

**Figure 6:**
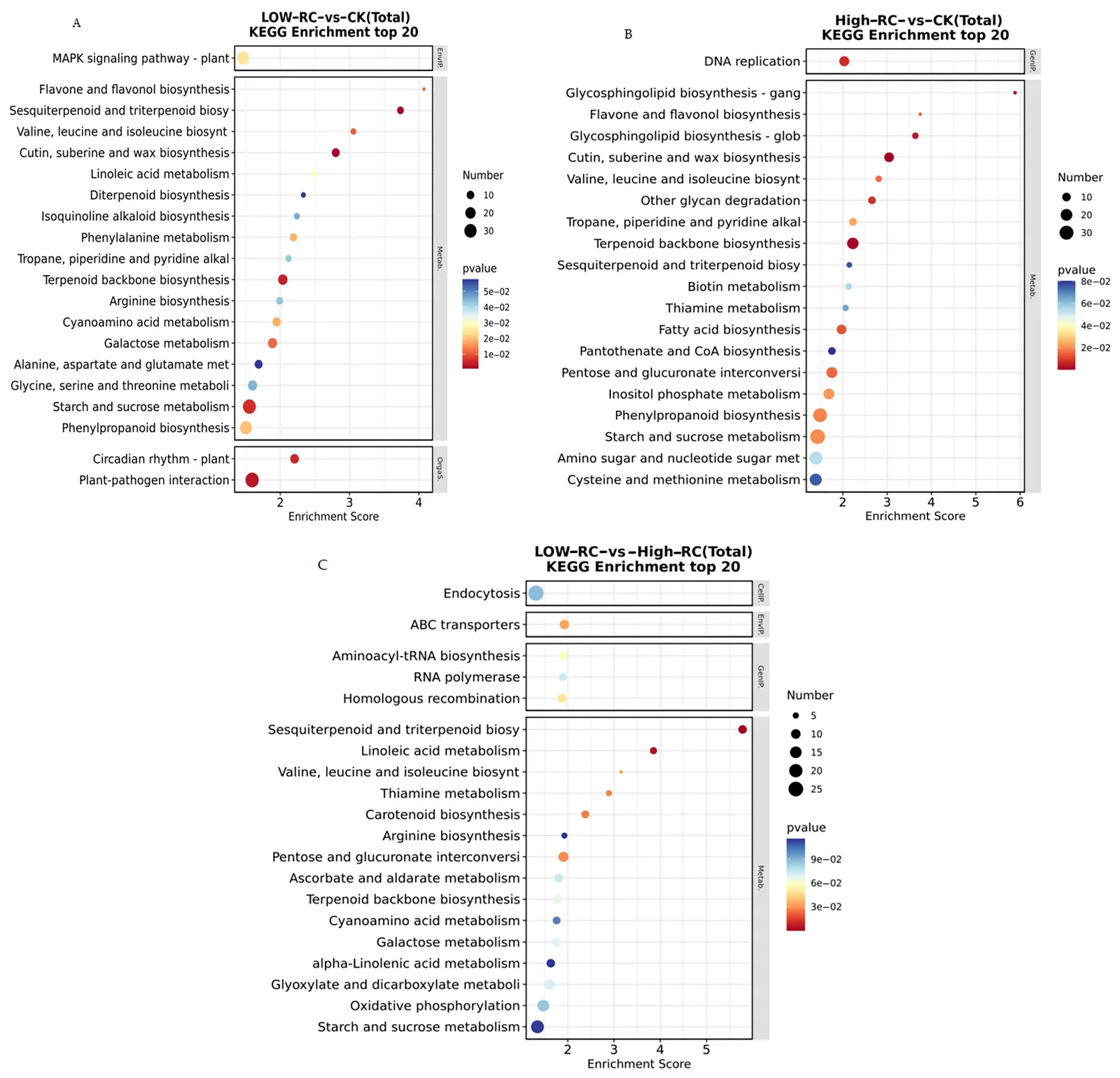
Enrichment analysis results of the KEGG pathway for differentially expressed genes: (**A**) Low_RC vs. CK; (**B**) High_RC vs. CK; (**C**) Low_RC vs. High_RC (in the bubble chart, color indicates enrichment significance and size indicates the number of enriched genes).

For the differentially expressed genes (DEGs) identified in each comparison group, we selected the top 20 significantly enriched KEGG pathways to generate enrichment bubble plots (Figure 6A–C). The results are as follows: In the Low_RC vs. CK comparison (Figure 6A), the significantly enriched pathways included the MAPK signaling pathway—plant, flavone and flavonol biosynthesis, sesquiterpenoid and triterpenoid biosynthesis, valine, leucine and isoleucine biosynthesis, and cutin, suberin and wax biosynthesis. Among these pathways, most exhibited high enrichment factors and extremely significant *p*-values, indicating that under low-dose imidacloprid treatment, significant transcriptional regulatory changes occurred in plant processes such as secondary metabolite synthesis, lipid metabolism, and amino acid synthesis.

In the High_RC vs. CK comparison (Figure 6B), the enriched pathways mainly included DNA replication, glycosphingolipid biosynthesis, flavone and flavonol biosynthesis, cutin, suberin and wax biosynthesis, and valine, leucine and isoleucine biosynthesis. These results suggest that high-concentration imidacloprid significantly affected multiple biological processes in plants, including nucleic acid metabolism (e.g., DNA replication), glycolipid metabolism (e.g., sphingolipid synthesis), epidermal protective structure formation (e.g., cuticle and wax synthesis), and amino acid metabolism. This implied that plants had made comprehensive adjustments in various aspects to cope with intense chemical stress.

In the Low_RC vs. High_RC comparison (Figure 6C), the number of significantly enriched pathways was relatively small. These mainly included basic cellular processes such as endocytosis, ABC transporters, aminoacyl-tRNA biosynthesis, RNA polymerase, and homologous recombination, as well as metabolic pathways such as linoleic acid metabolism, carotenoid biosynthesis, and ascorbate and aldarate metabolism. The overall enrichment level was low, with relatively large *p*-values. This indicated that the transcriptomic differences between low- and high-concentration treatments were limited, and the DEGs were mainly involved in some basic metabolic maintenance and stress response processes.

In conclusion, the KEGG enrichment analysis of the three comparison groups demonstrated that the DEGs of *A. membranaceus* var. *mongholicus* under imidacloprid stress were mainly involved in pathways such as plant secondary metabolite biosynthesis, lipid metabolism, amino acid metabolism, signal transduction (e.g., MAPK cascade), and antioxidant defense. This indicated that *A. membranaceus* var. *mongholicus* adapts to different concentrations of imidacloprid stress by coordinating multiple metabolic networks and signaling pathways, thereby achieving the re-regulation and balance of physiological functions.

### 3.3. Metabolomic and Transcriptomic Association Analysis

#### 3.3.1. Hierarchical Clustering and Dynamic Tree Cutting Module Analysis for the Construction of *A. membranaceus* var. *mongholicus* Weighted Metabolite Co-Abundance Network

**Figure 7:**
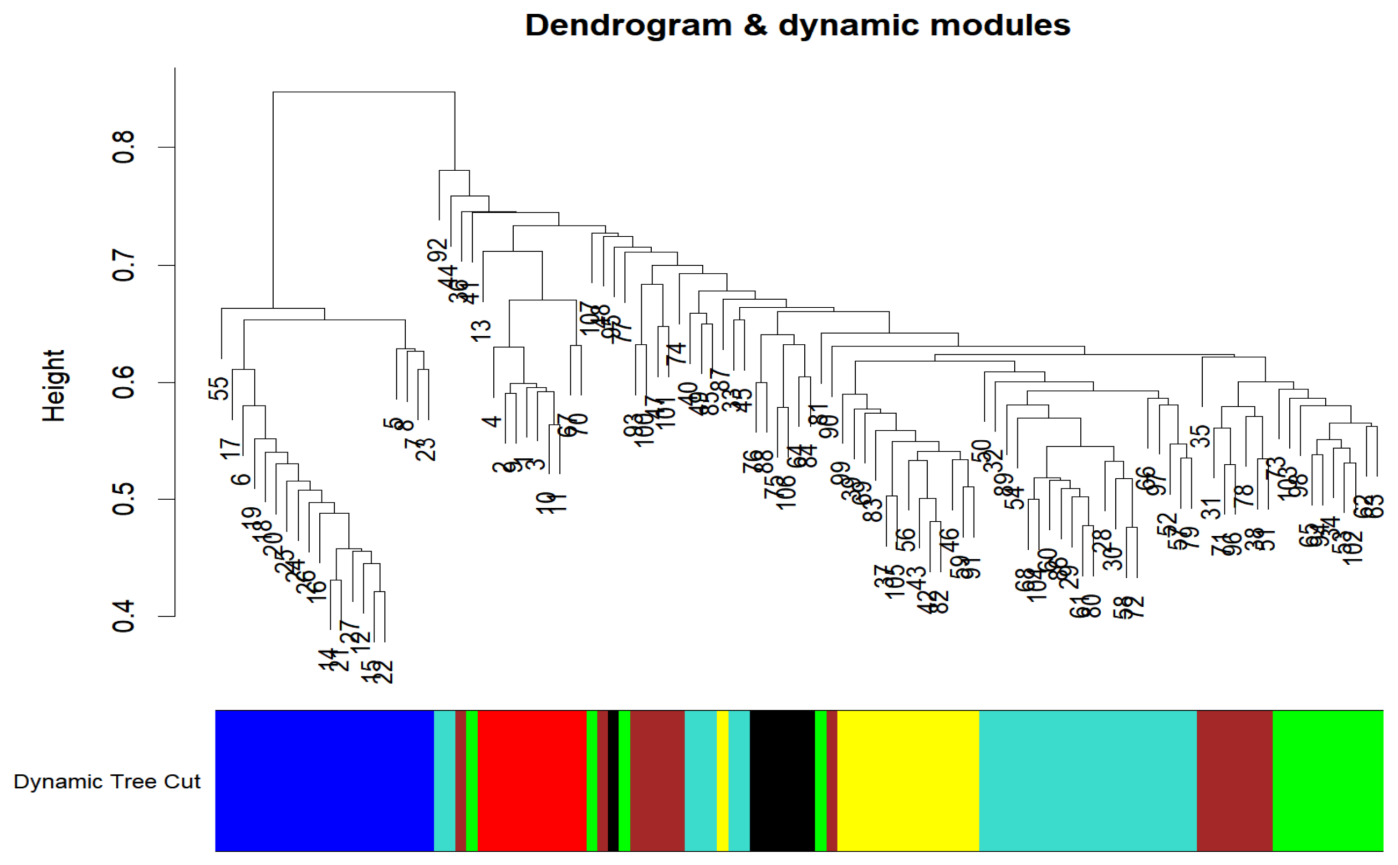
Tree clustering and dynamic module division of *A. membranaceus* var. *mongholicus* gene co-expression modules.

Weighted Metabolite Co-abundance network analysis (WMCNA) with the Dynamic Tree Cut algorithm revealed that the 80 target metabolites could be divided into 7 distinct co-expression modules, designated as black, blue, brown, green, red, turquoise, and yellow (Figure 7). The module memberships (kME values) of metabolites within each module were generally higher than 0.5, and those of the core metabolites exceeded 0.9. These results indicate that the module division exhibited good coherence and biological rationality. Combined with the correlation analysis between module eigengenes and gene significance (GS) under different treatment conditions, each module displayed distinct functional differentiation and specific environmental responsiveness.

The red module was identified as the core enrichment module for flavonoids and jasmonic acid (JA) signaling-related metabolites. Among these, the kME values of flavonoid glycosides such as luteolin-7-O-glucoside (kME = 0.985) and kaempferol-3-O-glucoside (kME = 0.962), as well as key components of the JA pathway including JA, JA-Ile, and OPDA, all exceeded 0.94. Furthermore, all metabolites in this module showed a strong positive correlation with the GS values under low-concentration treatment (GS_Low: 0.597–0.855), but only a weak or negative correlation with those under high-concentration treatment (GS_High). These findings suggest that the red module serves as a core functional unit in plant responses to low-concentration stress, and flavonoids may exert antioxidant and stress-defensive effects by synergizing with the JA signaling pathway. In contrast, the blue module specifically enriched isoflavonoids (daidzein, kME = 0.955; genistein, kME = 0.915), saponins (astragaloside II, kME = 0.969; saponin E, kME = 0.954), and antioxidant metabolites (glutathione, GSH, kME = 0.920; cysteine, kME = 0.897). The GS_High values of this module ranged from 0.654 to 0.990 and showed a significant negative correlation with GS_Low values (−0.429 to −0.024). This indicated that the blue module is a specific response module to high-concentration treatment, and isoflavonoids and saponins may synergistically participate in the maintenance of physiological homeostasis and the formation of medicinal quality under severe stress conditions.

The turquoise module and yellow module were predominantly composed of unknown metabolites (e.g., Metab_53, Metab_15). The kME value of Metab_53 in the turquoise module reached 0.975, and that of Metab_15 in the yellow module was 0.978. Both modules exhibited a moderate positive correlation with GS_Low (0.246–0.805) and a negative correlation with GS_High (−0.440 to −0.008). It is thus hypothesized that these modules may contain novel functional metabolites involved in basic metabolism or cross-stress responses. The green module, brown module, and black module had complex metabolite compositions, including amino acid derivatives, lipids, and unknown secondary metabolites, with kME values concentrated between 0.700 and 0.969. Their GS correlations showed diverse characteristics, suggesting that these modules may be involved in the synergistic regulation of metabolic networks or the maintenance of basic physiological processes.

In summary, the module division via WMCNA clearly defined the co-expression patterns of different metabolites. The specific correlations between the core modules (red and blue) and different treatment conditions provide an important theoretical basis for subsequent functional annotation of metabolites and identification of key components and regulatory pathways in stress responses, as well as laying a solid data foundation for the targeted improvement of plant stress resistance or medicinal quality traits.

#### 3.3.2. Co-Expression Network Analysis of Key Functional Genes (CHS/KCS/MYC2/PAL/ABCG40) in *A. membranaceus* var. *mongholicus*

**Figure 8:**
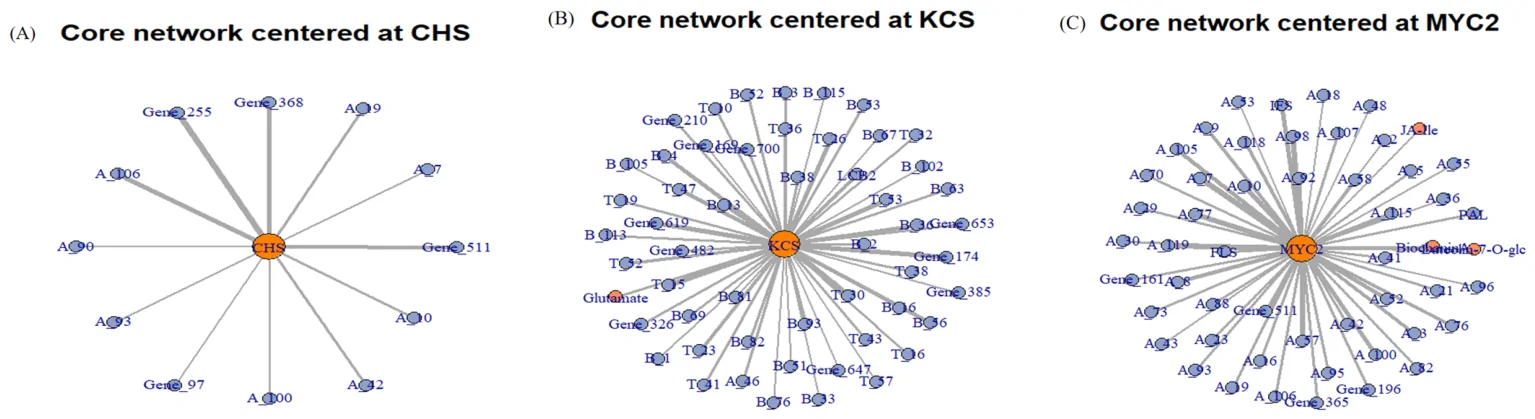

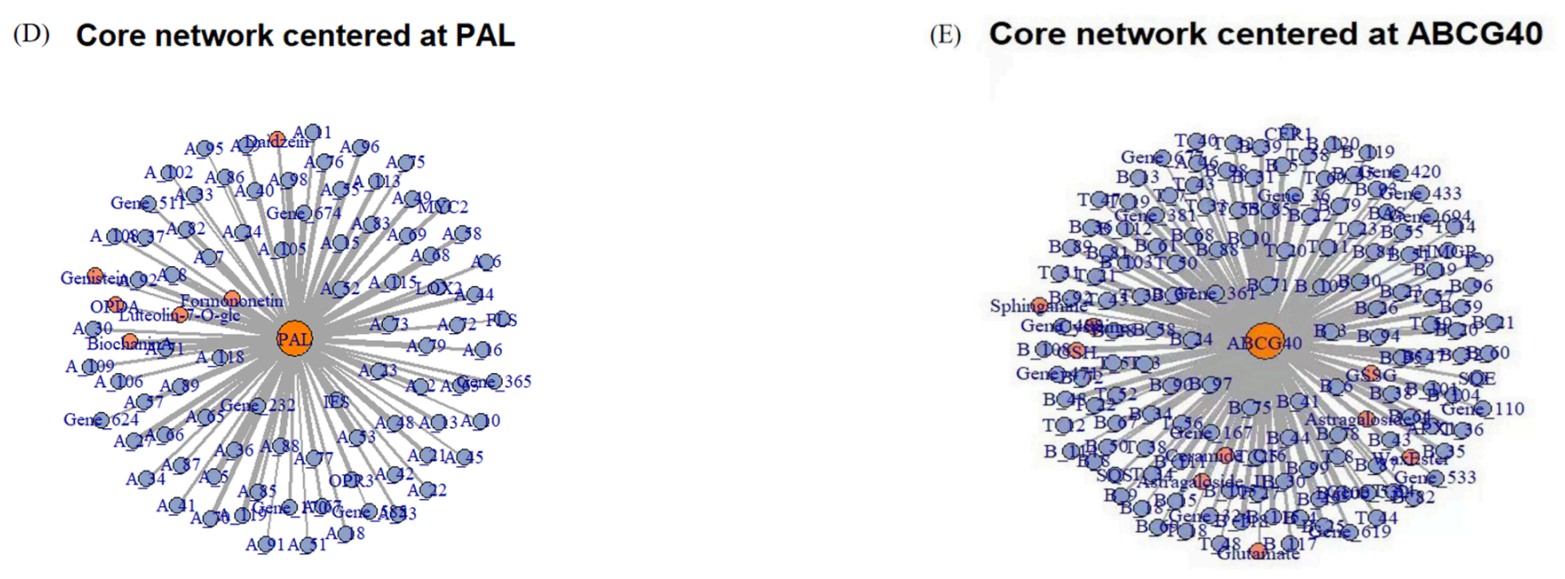
Co-expression network diagram of key functional genes of *A. membranaceus* var. *mongholicus*. Note: (**A**) chalcone synthase (CHS) co-expression network; (**B**) β-ketoacyl-CoA synthase (KCS) co-expression network; (**C**) regulatory network of the core transcription factor MYC2 in jasmonic acid signaling; (**D**) phenylalanine ammonia-lyase (PAL) co-expression network; (**E**) ABCG40 transporter co-expression network.

Integrated transcriptomic and metabolomic analyses were performed on *A. membranaceus* var. *mongholicus*. Multiple hub genes with annotated functions were screened based on correlation-based co-expression networks. These genes exhibited significant correlations with downstream genes and metabolites and may be implicated in processes associated with plant stress responses (Figure 8A–E). Chalcone synthase (*CHS*), a key rate-limiting enzyme in flavonoid biosynthesis, displayed a typical hub-and-spoke topological structure within the co-expression network (Figure 8A). The *CHS* gene was significantly co-expressed with numerous transcripts (e.g., *Gene255*, *Gene368*, *Gene511*) and directly correlated with several metabolite nodes, including A90, A95 and A96. This suggests that *CHS* may participate in flavone skeleton formation and have close correlative links to the accumulation and modification of downstream flavonoid glycosides. Notably, *CHS* showed strong correlations with unknown metabolites A0 and A2, implying that this gene may possess novel potential roles in the biosynthesis of flavonoid derivatives unique to *A. membranaceus* var. *mongholicus*. The correlative patterns in the network preliminarily reflect the central correlative position of *CHS* in flavonoid biosynthesis of this species, which can serve as a reference for further dissection of metabolic correlation patterns underlying medicinal quality formation in *A. membranaceus* var. *mongholicus*.

β-Ketoacyl-CoA synthase (*KCS*), the rate-limiting enzyme for very-long-chain fatty acid (VLCFA) biosynthesis, occupied a core correlative position in the lipid metabolism network (Figure 8B). Co-expression network results reveal significant co-expression between *KCS* and multiple lipid-metabolism-related genes (series B nodes) as well as membrane transporter genes (series T nodes). This indicates that *KCS* may participate in VLCFA biosynthesis and bear potential correlative associations with cuticular wax formation on the root epidermis and membrane lipid metabolism in *A. membranaceus* var. *mongholicus*. Moreover, *KCS* showed strong correlations with several unannotated genes, such as *Gene210*, suggesting its possible involvement in synthesizing lipid derivatives characteristic of *A. membranaceus* var. *mongholicus*. The network features preliminarily uncover the potential central correlation of *KCS* in lipid metabolism and stress response, offering novel insights into the root development and environmental adaptation mechanisms of this medicinal plant.

As the core transcription factor of jasmonic acid (JA) signaling, *MYC2* formed a highly complex central correlation hub in the secondary metabolism network of *A. membranaceus* var. *mongholicus* (Figure 8C). Its co-expression network contained abundant stress-responsive genes (nodes prefixed with AQ) and secondary metabolic genes, implying that *MYC2* may integrate JA signal transduction and present synergistic correlations with flavonoid biosynthesis and plant stress responses. A hierarchical correlative pattern was observed in the network, which suggests that *MYC2* may form multi-layered associative modules with downstream *bHLH* transcription factors and metabolic enzymes via cascade correlations. These observations reflect the key hub features of *MYC2* in the “stress–metabolism” correlative pathway, providing data support for future research on JA-mediated correlation patterns governing *A. membranaceus* var. *mongholicus* quality formation.

Phenylalanine ammonia-lyase (PAL), the initiating enzyme of the phenylpropanoid pathway, exhibited distinct modular properties in its co-expression network (Figure 8D). The network contained a large set of genes involved in branched phenylpropanoid pathways (AQ-prefixed nodes) and showed tight correlations with key flavonoid biosynthetic enzymes (CHS, CHI) and lignin-related genes. Such correlative features suggest that PAL may modulate phenylpropanoid metabolic flux and maintain close correlative associations with flavonoid and lignin biosynthesis in *A. membranaceus* var. *mongholicus*. Meanwhile, multi-tiered upstream gene correlations were detected in the *PAL* network, indicating coordinated expression between PAL and various transcription factors and preliminarily demonstrating its key hub property across the global secondary metabolic network.

As a member of the ATP-binding cassette (ABC) transporter G family, *ABCG40* acted as a hub in the metabolite transport network (Fig. E). Co-expression analysis revealed that ABCG40 was significantly co-expressed with multiple membrane transporters (e.g., *B_008SH_B024*, *B_96_152_BQ9BQ7*) and glycosylation-related genes (e.g., *TQ2_BQ9BQ4_36*), and also connected to several species-specific genes (e.g., *Astraglb_sa_6h_Gene110*). This implies that *ABCG40* may participate in transmembrane translocation and intracellular distribution of secondary metabolites, with potential correlative links to the accumulation of bioactive constituents in *A. membranaceus* var. *mongholicus*. The network results preliminarily reveal the vital correlative characteristics of *ABCG40* in secondary metabolite transport, providing new clues to unravel potential associative mechanisms coupling biosynthesis and compartmental distribution of medicinal ingredients.

#### 3.3.3. WGCNA-Based Multilevel Association Network of Genes–Metabolites–Traits

**Figure 9:**
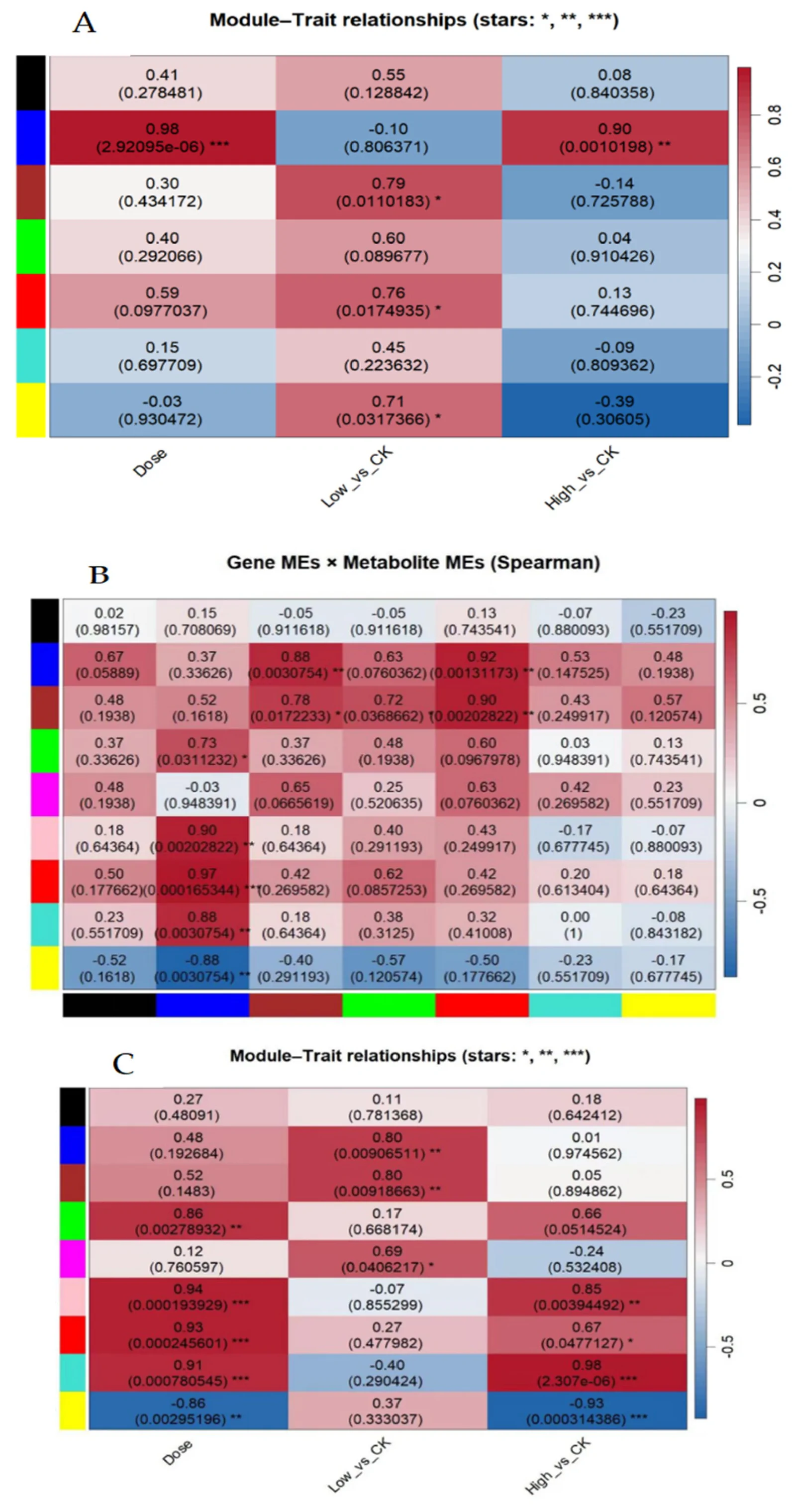
Multilevel association network of genes, metabolism and traits in *A. membranaceus* var. *mongholicus*: (**A**) association network of gene co-expression modules and phenotypic traits (Low_RC vs. CK, High_RC vs. CK); (**B**) module–trait correlation expansion; (**C**) Spearman correlation network of gene modules and metabolite modules. * *p* < 0.05, significant correlation; ** *p* < 0.01, highly significant correlation; *** *p* < 0.001, extremely significant correlation. The red color indicates positive correlation, while blue color represents negative correlation.

Using weighted gene co-expression network analysis (WGCNA), we constructed an association network between gene co-expression modules and phenotypic traits in *A. membranaceus* var. *mongholicus* (Figure 9A). The results show that the Eblue module exhibited an extremely significant positive correlation with the Low_RC vs. CK treatment (r = 0.98, *p* < 0.001) and a significant positive correlation with the High_RC vs. CK treatment (r = 0.90, *p* < 0.01), suggesting that this module may be involved in the regulation of basal metabolism in *A. membranaceus* var. *mongholicus* under non-stress conditions. In addition, the brown module was significantly positively correlated with High_RC vs. CK (r = 0.79, *p* < 0.05), whereas the yellow module showed a negative correlation trend with High_RC vs. CK (r = −0.39). These findings revealed potential functional divergence among different modules during the response of *A. membranaceus* var. *mongholicus* to environmental stress. The above results preliminarily identify core regulatory modules closely associated with the ecological adaptability of *A. membranaceus* var. *mongholicus*.

In an extended module–trait association analysis, the pink module (r = 0.94, *p* < 0.001), the red module (MEred, r = 0.93, *p* < 0.001), and the turquoise module (r = 0.91, *p* < 0.001) were all found to have an extremely significant positive correlation with Low_RC vs. CK, while the turquoise module displayed the strongest negative correlation with High_RC vs. CK (r = −0.93, *p* < 0.001) (Figure 9B). Notably, both the Eblue and brown modules were significantly positively correlated with High_RC vs. CK (r = 0.80, *p* < 0.01 for each), indicating that these modules may synergistically participate in the response of *A. membranaceus* var. *mongholicus* to high stress. The negative correlation pattern of the yellow module further confirmed its potential role as a repressive regulatory factor in stress responses.

Subsequent Spearman correlation analysis between gene modules and metabolite modules uncovered a multilevel regulatory network (Figure 9C): gene modules including Eblue and brown were significantly positively correlated with multiple metabolite modules (e.g., eblue and metabolite module 3, r = 0.88, *p* < 0.01), demonstrating a synergistic relationship between transcriptional regulatory changes and metabolite accumulation; the MEred module showed an extremely significant positive correlation with metabolite module 2 (r = 0.97, *p* < 0.001), suggesting that this gene module may directly regulate the biosynthesis of secondary metabolites such as flavonoids; in contrast, the yellow module was negatively correlated with several metabolite modules (e.g., metabolite module 2, r = −0.88, *p* < 0.01), further verifying its potential role as a repressive regulatory node in stress responses.

## 4. Discussion

This study integrated transcriptomic and untargeted metabolomic analyses to reveal the molecular and metabolic responses of *A. membranaceus* var. *mongholicus* seedlings to short-term imidacloprid stress at two exposure concentrations. Rather than merely describing phenotypic omics changes, the present study focused on interpreting the regulatory crosstalk between pesticide-induced stress signaling and secondary metabolic reprogramming, as well as the concentration-dependent adaptive strategies adopted by medicinal plants under agrochemical stress.

Secondary metabolism serves as a core defensive strategy for plants coping with abiotic stress. Consistent with previous studies on plant pesticide responses [34,35], imidacloprid exposure markedly promoted the accumulation of flavonoids and saponins, the signature bioactive components of *A. membranaceus* var. *mongholicus*. The enhanced biosynthesis of these antioxidant metabolites indicated that the seedlings initiated chemical defense systems to mitigate pesticide-induced oxidative damage. Meanwhile, the altered accumulation of terpenoids and sterols further implied the remodeling of membrane structure and stress-related signal transduction under imidacloprid treatment [36]. Such metabolic alterations were coordinately supported by transcriptomic evidence, in which defense- and secondary metabolism-related genes were predominantly up-regulated, confirming that transcriptional activation constitutes the core genetic strategy for plant stress adaptation [37,38].

Concentration-dependent response patterns were clearly observed in both the transcriptomic and metabolomic profiles. Low-concentration imidacloprid primarily activated JA signaling, calcium transduction, MAPK cascades, and flavonoid biosynthesis pathways, which represent a typical mild defense response enabling rapid stress perception and metabolite adjustment [39,40,41]. Comparatively, high-concentration stress triggered more extensive transcriptional remodeling involving DNA replication, nucleic acid metabolism, and cell wall reconstruction, reflecting aggravated cellular stress and genome-level perturbation under severe pesticide exposure [42,43]. Although transposon-related pathways were enriched under high-dose stress, such enrichment only reflects transcriptional alteration; definitive genomic instability and transposon activation require further experimental validation, consistent with the inherent limitations of omics correlation analysis [44,45].

Multi-omics co-expression network analysis further uncovered the core regulatory modules linking stress signaling and metabolite accumulation. Consistent with reported plant secondary metabolic regulatory mechanisms [46,47,48], key rate-limiting enzymes *CHS* and *PAL* were identified as central hubs governing flavonoid metabolic flux under stress. The JA-responsive transcription factor *MYC2* exhibited extensive co-expression with downstream defense and metabolic genes, demonstrating that JA signaling acts as a pivotal upstream module integrating pesticide stress signals and secondary metabolic reprogramming in *A. membranaceus* var. *mongholicus* [40,49,50,51]. Moreover, the transporter gene *ABCG40* was centrally located in the network, suggesting that coordinated synthesis and transmembrane transport of secondary metabolites contribute to the establishment of plant chemical defense systems [52,53]. These multi-omics associations provide novel mechanistic insights into how medicinal plants balance stress resistance and active ingredient accumulation under agrochemical stress.

Based on the overall omics alterations observed in this study, we propose a hypothetical “damage–defense–adaptation” staged response model to systematically interpret the dynamic adaptation process of *A. membranaceus* var. *mongholicus* under imidacloprid stress. Pesticide exposure first induces oxidative stress damage; subsequently, plants activate multi-signal pathways to enhance antioxidant defense and protective metabolite accumulation; finally, plants rebalance growth-defense resource allocation to achieve potential physiological homeostasis [24,54,55,56]. Importantly, this model is a theoretical hypothesis derived solely from transcriptomic and metabolomic correlation data, and further physiological evidence is required for validation.

Collectively, appropriate imidacloprid dosage can moderately activate secondary metabolism and promote the accumulation of medicinal active ingredients, whereas excessive pesticide application induces intense cellular stress and abnormal transcriptional reprogramming. These findings provide theoretical guidance for standardized pesticide application during *A. membranaceus* var. *mongholicus* cultivation to balance pest control and medicinal quality maintenance.

Notably, this study contains several unavoidable limitations. First, only short-term 24 h stress treatment and leaf tissue sampling were performed, lacking long-term dynamic monitoring and tissue-specific verification. Second, all conclusions are derived from omics correlation analysis, without physiological index validation to confirm stress intensity and adaptive phenotypes. Third, no imidacloprid residue detection was conducted, and core medicinal components were not quantitatively determined via targeted metabolomics. Future studies combining physiological phenotyping, residual detection, targeted metabolite quantification, and gene functional verification are essential to further validate the regulatory mechanisms proposed in the present study.

## 5. Conclusions

In this study, integrated transcriptomic and untargeted metabolomic analyses revealed early concentration-dependent responses of *A. membranaceus* var. *mongholicus* seedlings to imidacloprid exposure. Both recommended-dose and high-dose treatments altered the accumulation of flavonoids, saponins, terpenoids, amino acid derivatives, and energy-related metabolites and induced extensive changes in genes associated with secondary metabolism, stress signaling, metabolite transport, and cellular remodeling. Integrated network analysis identified *CHS*, *PAL*, *MYC2*, *KCS*, and *ABCG40* as candidate regulators linking imidacloprid-induced stress responses with secondary metabolite biosynthesis and transport. These findings provide candidate pathways and genes for future functional validation and offer a molecular basis for evaluating pesticide-stress responses in medicinal plants. Long-term cultivation experiments, physiological measurements, pesticide residue analysis, and targeted quantification of bioactive compounds are needed before drawing conclusions about yield, safety, or medicinal quality.

## Figures and Tables

**Table 1:** Statistics on sequencing data quality.

Sample	CleanReads (M)	CleanBases (G)	%≥30	GC Content
A1	46.58	6.8	93.56	43.36
A2	46.43	6.8	92.81	43.1
A3	45.94	6.71	93.45	43.07
B1	50.18	7.36	93.37	43.26
B2	50.44	7.42	90.88	42.93
B3	48.46	6.92	93.68	43.62
CK1	47.66	6.94	92.85	43.12
CK2	48.98	7.21	93.02	43.1
CK3	50.01	7.35	93.5	43.17

Note: Clean Reads refers to the total number of clean data reads; Clean Bases refers to the total number of clean data bases; %≥Q30 refers to the proportion of bases with a quality value ≥ 30; GC Content refers to the percentage of GC content.

**Table 2:** Statistics of gene function annotation results.

Database	Number of Genes	Percentage
NR	35,207	64.31%
Swissprot	24,991	45.65%
KEGG	6932	12.66%
KOG	19,735	36.05%
eggNOG	31,467	57.48%
GO	21,962	40.11%
Pfam	21,347	38.99%
Annotated in all databases	19,067	34.83%
Total unigenes	54,748	100%

Note: Annotated denotes the number and percentage of genes annotated in the corresponding database.

## Data Availability

The data are available from the first author upon reasonable request.
